# Monitoring and stability analysis of roadbed & high slope prior to highway construction

**DOI:** 10.1371/journal.pone.0303860

**Published:** 2024-06-17

**Authors:** Rengye Zhao, Shengliang Cao, Bowen Ni, Zhao Liu

**Affiliations:** 1 CCCC First Highway Consultants Co., Ltd., Xi’an, Shaanxi, China; 2 School of Highway, Chang’an University, Xi’an, Shaanxi, China; Baba Ghulam Shah Badshah University, INDIA

## Abstract

Monitoring in highway projects is significant for the safety, efficiency and quality of construction. This paper proposes a detailed method for monitoring soft-soil roadbed and high-slope, and the layered settlement gauge and total station are employed to carry out experimental monitoring. The law and stability of soft-soil roadbed settlement and deformation under high-slope are further analyzed. The results show that the cumulative values of roadbed settlement and slope platform deformation in general both increase with the increase of monitoring time. However, near 180 days, an abnormal settlement phenomenon was monitored on both sides of the highway with a maximum value of 9.44 mm. This phenomenon was captured at gauge #1, #3, and #4 on observation stake 3, and exact oppositely, it was also observed at gauge #2 on observation stake 4. Moreover, unusual deformations of the high-slope platforms occurred over a period of 10 to 30 days, and these unusual settlements and deformations are indicative of the highway’s instability. Therefore, the monitoring on soft-soil roadbed settlement and high-slope deformation can provide reference for highway construction.

## Introduction

Monitoring and stability analysis of roadbed and slope have been the major focus on geotechnical engineering [1, 2]. The complexity of soft soil roadbeds and high slopes in highway construction is not only manifested in the lithology, primary structure and spatial combination, it is also expressed in the internal and external geologic stresses, especially in engineering activities such as highway excavation, slag heap, drainage and so on [3, 4].

Current research in roadbed monitoring is essentially engineering-dependent. Ma et al. [5] monitored and analyzed the temperature of a section for roadbed on Tibetan Plateau, and they proposed three measures to regulate and cool the roadbed, which are useful for protecting the permafrost underneath the roadbed. Antonovskaya et al. [6] presented a potential and broadband seismograph-based approach to roadbed condition monitoring, however, this way is passive since it uses vibrations from passing trains for roadbed testing. Li et al. [7] used FLAC2D to simulate soft soil roadbeds and conducted on-site monitoring of roadbed deformation under the project of K28+830 of Hangzhou Expressway test section, and they pointed out that injected cement mixing piles can strengthen the stability of soft soil roadbed. Chen et al. [8] established embankment safety monitoring techniques and early warning systems for overall roadbed stability and pavement performance. Gou et al. [9] and Zhang et al. [10] concentrated on the implementation method of roadbed and pavement compaction technology in road construction, and the proposed roadbed compaction technology has guidance for road engineering. Liu et al. [11] described the common diseases and prevention techniques related to roadbeds in highway construction, and they suggested that the implementation of diversified techniques for highway construction can improve the stability and safety of roadbed structures. Ye et al. [12] presented three common roadbed engineering disorders as examples of actual projects, and they found that pavement smoothness accounted for the largest part of 30%, which demonstrated the importance of maintenance for highway roadbeds.

There also have been some studies on slope monitoring. Ohnishi et al. [13] developed a digital photogrammetry-based monitoring technique, which was used to reproduce slope shapes with a high degree of accuracy from numbers of images taken in all directions. Yadav et al. [14] describe the design and implementation of a real-time, cost-effective wireless system for monitoring slopes, which even has an early warning function. Xu et al. [15] proposed a fiber Bragg grating (FBG) inclination sensor for slope monitoring, which has the advantages of high accuracy and small size. Vanneschi et al. [16] explored the possibility of implementing slope monitoring and risk management programs by using remote sensing techniques and conventional surveying equipment. Ding et al. [17] reviewed geotechnical instrumentation used for slope monitoring in the Australian surface mining industry, and then described an automated slope monitoring system based on the integration of electronic geotechnical instrumentation with specialized software. Wang et al. [18] conducted a three-dimensional numerical simulation by using the strength discount method to further analyze the 3D deformation, and damage characteristics of the Lijiazhai slope on the Shicheng-Jian highway in Jiangxi Province is further analyzed. Gu et al. [19] developed a novel early warning system for monitoring road slopes, and the results of numerical simulations performed showed that the system was within 15% error. Lee et al. [20] explored the slope breaching mechanism of highway by studying Provincial Highway 24, and they evaluated the potential slope sliding behavior based on theoretical and tectonic-geological data. Yilmaz et al. [21] proposed a novel method for calculating the slope angle of highway by using topographic and geological datasets in a GIS environment to determine the appropriate slope of a road, and they elaborated on the application of the method using a highway in Turkey as an example. Maduka et al. [22] pointed out that road base materials, geology and construction materials affect the quality of highway pavements, and they assessed the potential for slope failure by road damage and slope monitoring along the Nsukka-Adoru-Idah Expressway.

Reviewing the aforementioned literature, most of the studies on roadbed and slope monitoring seem to focus on the development of the system or on the realizability of some methods. Actually, for the preliminary work of highway construction, it is more important to realize the monitoring of roadbed and highway slope by using various kinds of measuring instruments. Therefore, this paper will rely on the engineering construction of Jiuzhi-Malkang highway, and propose a detailed method for roadbed and highway slope monitoring, including the selection, arrangement and test program of measuring instruments.

## Geographic features of the Jiuzhi-Malkang highway

This work is an engineering construction project and which is permitted by Sichuan Juma Expressway Group Co., Ltd. And the study is also exempt from institutional ethics committee approval.

**Fig 1:**
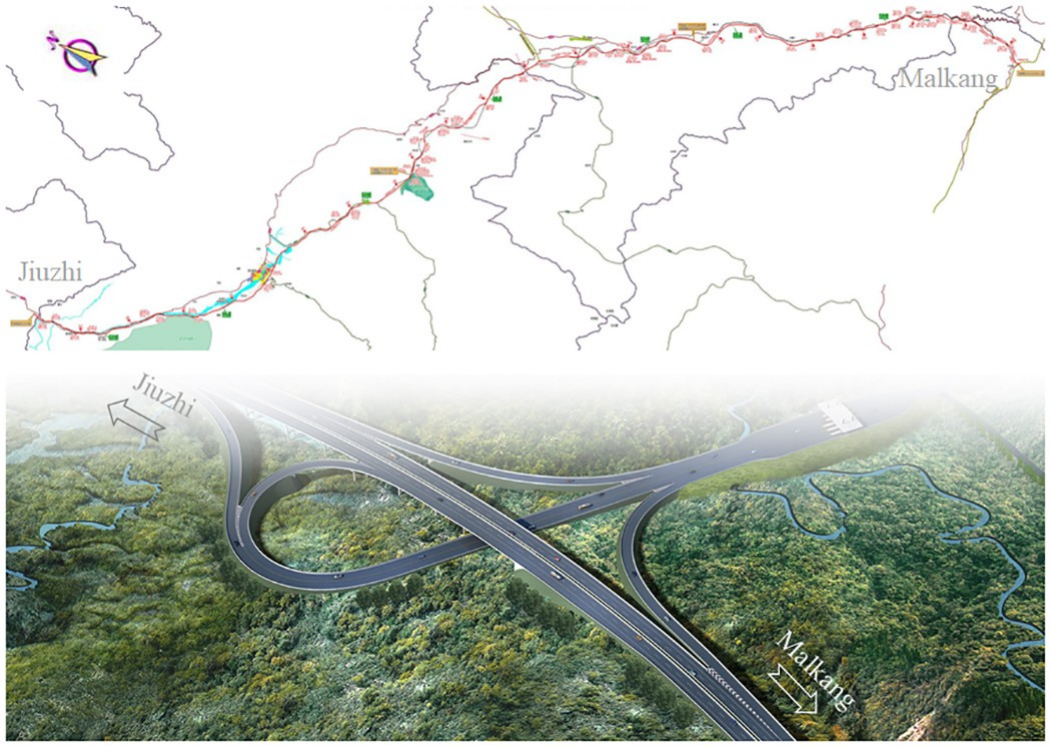
Two-dimensional layout of the Jiuzhi-Malkang highway and part of the completed section of the highway.

As an important part of G0615 Delingha-Malkang, Jiuzhi-Malkang highway is an outstanding component of the national highway network. It can be seen from Fig 1 that, the project adopts technical standards in sections: the starting point to Jihui, Haizishan to Brush Temple section take 100 km/h designed speed standard, with roadbed width of 26.0 m, mileage of 166.373 km; Jihui to Haizishan, Brush Temple to the end of the section use 80 km/h designed speed standard, with roadbed width of 25.5 m, mileage of 52.703 km. The construction of Jiuzhi-Markang highway is of great significance in improving the national highway network and regional road network structure, which accelerates the construction of the western comprehensive transportation hub and promotes the coordinated development of the region.

The central part of Jiuzhi-Malkang highway is situated in an area characterized by gentle, open and slight cuts, which makes it form a high plain and hilly terrain. The poor water drainage and swamp development lead to the widespread weak foundations in this area. A special kind of peat soil composed of sediments is developed in the swamp area, which is featured by high compressibility and low mechanical strength, and it is very easy to develop into a weak foundation. Moreover, the clay accumulated between gullies and valleys shows plastic or even soft plastic shape under the action of groundwater and surface water bodies, and the groundwater is blocked by these clays, which leads to poor drainage, and weak foundations can also be formed in part of the gullies and valleys. Weak foundations are generally recognized as having high water content, low bearing capacity, large compressive deformation and easy to yield uneven settlement [23]. To facilitate highway construction, it is necessary to modify and handle these soft foundations to form soft soil roadbeds. For different thicknesses of soft foundations and structures, treatments are carried out using the methods shown in Table 1.

**Table 1:** Treatment for soft foundations and structures of different thicknesses.

Weak foundation thickness	Treatment	Specific measure
*h* < 4 m	Shallow treatment	Refill, Drainage, Rock dredging [24]
4 < *h* < 15 m	Thick treatment	Crushed stone piles, plain concrete piles [25]
*h* > 15 m	Thick treatment	Prestressed pile [26]

The route of Jiuzhi-Malkang Highway repeatedly crosses mountains and spreads out on mountain slopes, which have steep natural cross-slopes, generally ranging from 20° to 35°, with the steepest being more than 45°. Because of topography, there are many rocky high slopes with the height greater than 30 m, and this kind of terrain with a large inclination angle and high height is called high slope. However, the slope surface is prone to collapse and falling blocks, and the mudstone will easily be weathered and softened after slope excavation. So it is required to carry out slope protection in time. As opposed to the treatment of soft soil roadbeds shown in Table 1, protective measures are generally planting grass by hanging barbed wire.

## Monitoring techniques and test setup

### Soft soil roadbed

Monitoring of soft soil roadbed is not only able to master the rule of settlement change and lateral movement for foundation soil, it can also be used to further analyze the deformation and force of roadbed foundation. Which ultimately ensures the construction safety and quality in soft soil roadbeds with great significance.

**Fig 2:**
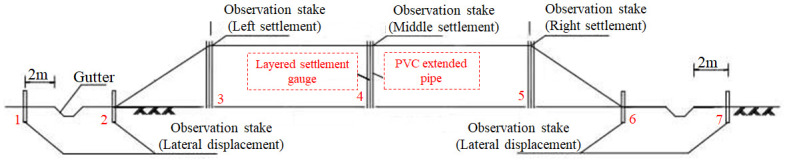
Layout scheme of observation stakes for soft soil roadbed.

The monitoring quantities of soft soil roadbed are mainly vertical displacement, vertical settlement and horizontal displacement. Considering the factors of topography, filling height and ancillary structures, seven stakes for observation are set on the soft soil roadbed section, which are shown in Fig 2. There are 4 observation piles for horizontal displacement, which are located at the outer edge of the side ditch (observation stakes 2 and 6) and 2 m away from the side ditch (observation stakes 1 and 7). While there are 3 observation points for settlement, which are placed at the highway’s centerline (observation stake 4) and both shoulders (observation stakes 3 and 5).

**Fig 3:**
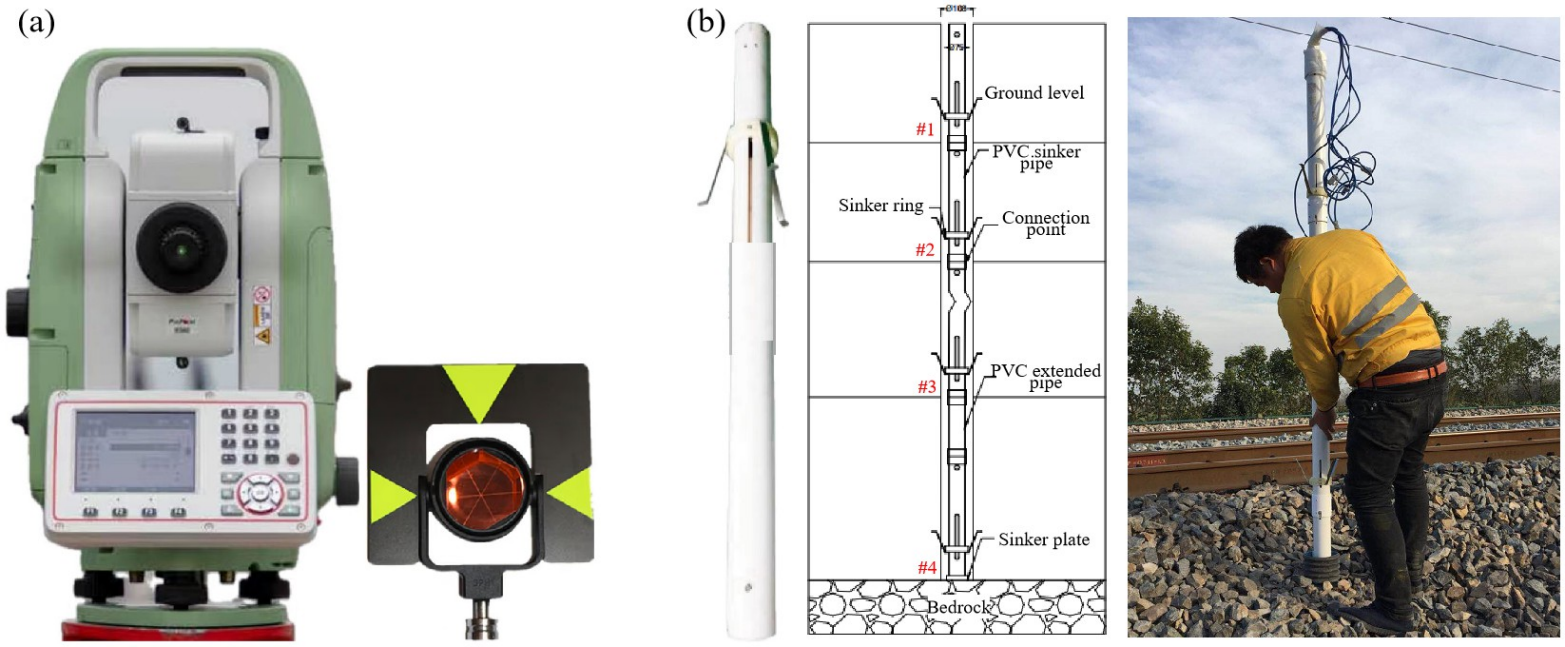
(a) Total station and accompanying prism for measuring horizontal displacements. (b) Layered settlement gauge, its structure and field installation for measuring vertical settlement.

Observation stakes on both sides of the highway are 20 cm20 cm precast concrete stakes, with 1.4 m buried depth and 0.1 m exposed height. The backfill around stakes is dense, and the upper 50 cm of the stakes are fixed with concrete grouting. The displacement information of these positions are measured and acquired by total station (Leica TZ08), which is given in Fig 3(a). The requirement for the location of all observation points is a stable, firm and gentle area. Moreover, in order to ensure the accuracy of monitoring, no less than three initial value tests are conducted for these monitoring points as a database, and the average of three stabilized values in database is taken as the original baseline data. Furthermore, as shown in Fig 3(b), vertical displacements are carried out by layered settlement gauges, which consist of four single-point settlement gauges (YT-DG-0200) (#1, #2, #3 and #4) connected in series by mounting kits, for performing layered settlement monitoring. When the layered settlement gauge is used for settlement observation, it is necessary to firstly determine the depth of each observation layer, and the four single-point settlement gauges are placed at the position should be monitored with PVC gauge tube, finally, the PVC gauge tube is filled with dry fine sand to ensure the function of continuous observation.

### High slope

Monitoring for the high and steep slope is an important means to verify the design and guide of construction, which can not only check the correctness of the parameters and design theory, also amend the design and construction methods timely by dynamic information feedback to determine and optimize the construction parameters. Monitoring for the high and steep slope is the key content of highway construction informationization.

Monitoring for the high and steep slope for highway projects is categorized into crack tracking and surface displacement measurement. The monitoring of slope surface cracks can be realized by manual observation. The main issues for monitoring can be summarized as follows: the presence of new cracks and landslides on the slope surface; enlargement and extension of existing cracks; fault misalignment; and uplift and tensioning at the slope footing.

**Fig 4:**
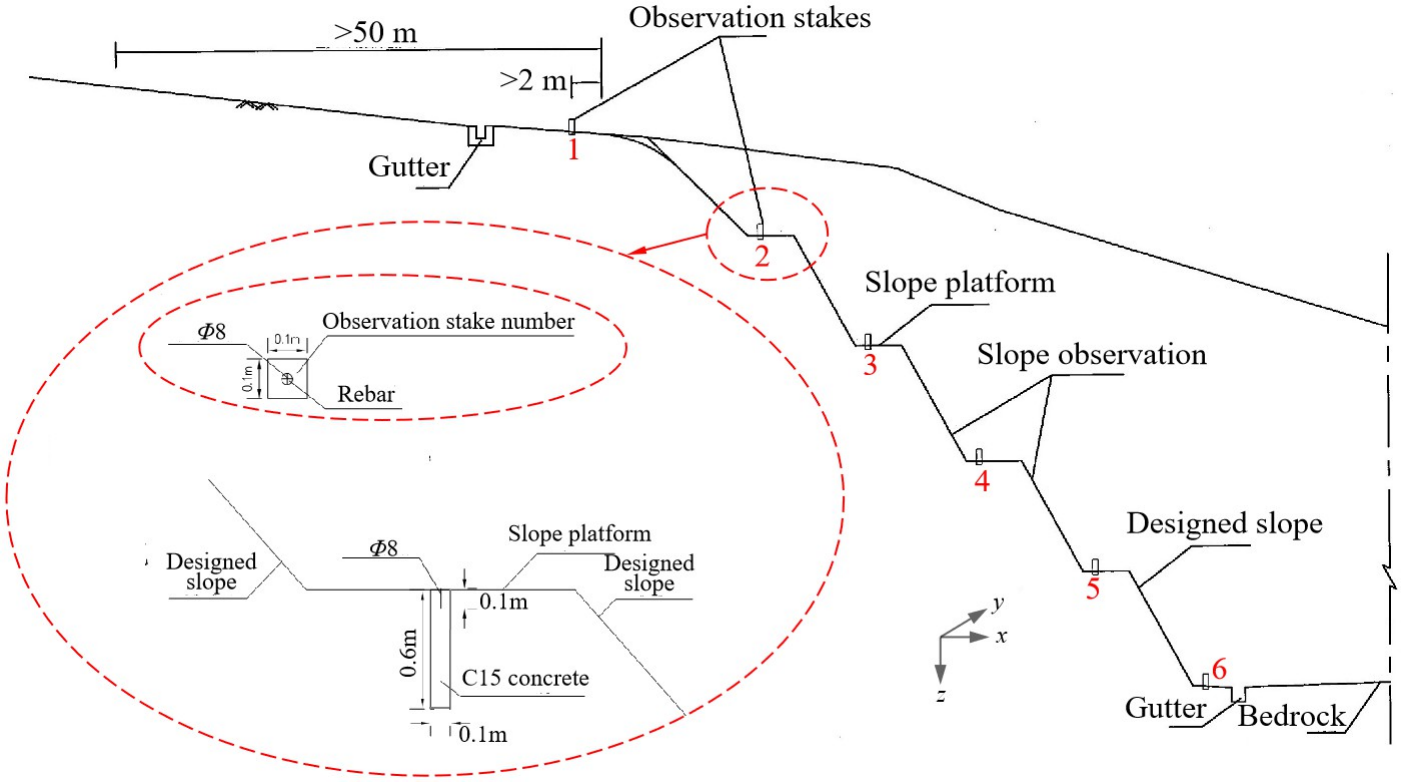
Schematic layout of observation stakes for high slopes.

As for the measurement of surface displacement, which is started from the highest point of excavated slope, the observation sections are arranged at slope’s topside (observation stake 1), each slope platforms (observation stakes 2, 3, 4, and 5) and slope’s foot (observation stake 6), as shown in Fig 4. Fixed piles (0.1 m0.1 m0.6 m) are buried at the monitoring points, and the vertical and horizontal displacements can be observed here by using the total station shown in Fig 3(a).

## Result analysis for monitoring and testing

In this section, the settlement data obtained from real-time monitoring, descriptive conditions and displacement trend changes in S1 File have been analyzed thoroughly.

### Analysis for soft-soil roadbed monitoring

**Fig 5:**
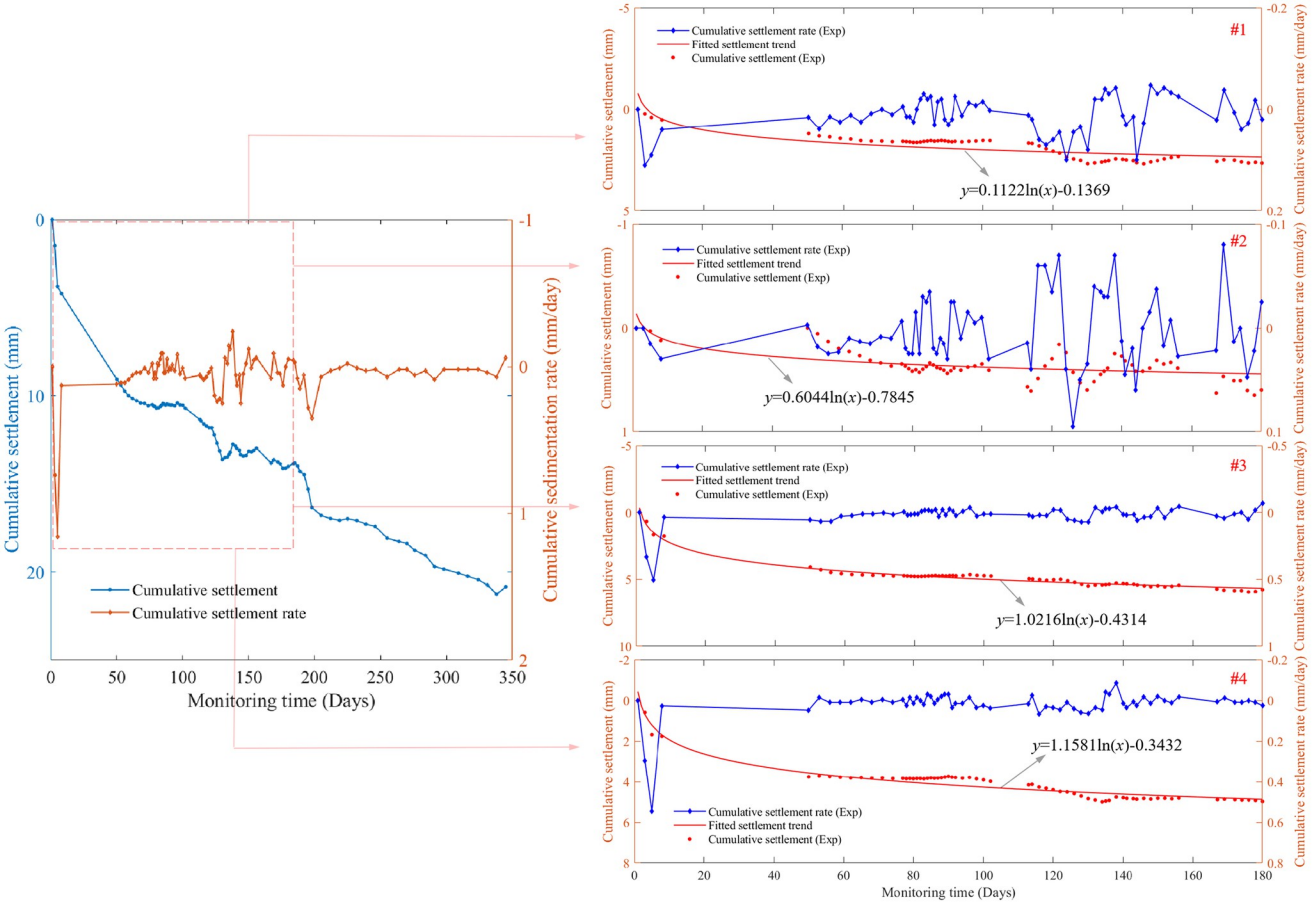
Cumulative settlement values and their rates obtained by the layered settlement gauge at observation stake 4 used for soft-soil roadbed monitoring. The left graph shows the cumulative values of the four settlement gauges, and the right graph shows the experimental data obtained by settlement gauges #1, #2, #3 and #4, from top to bottom, respectively.

The key parameter of soft-soil roadbed monitoring is the settlement of highway pavement, therefore, taking the K114+340 section in construction project of Jiuzhi-Malkang highway as an example, firstly, the position of observation stake 4 located at highway’s central axis shown in Fig 2 is tested, and the settlement displacements of each layer for soft-soil roadbed are obtained by using the layered settlement gauge, the results of long-term measurements are shown in Fig 5. Where, the left side of Fig 5 indicates the variation of cumulative settlement value and settlement rate, obtained by adding up the data collected by four settlement gauges. While the right side indicates the variation of observation data captured by each of the #1, #2, #3 and #4 settlement gauges, respectively. Moreover, according to the experimental data of the observed cumulative settlement, Gauss’s theorem is used to generate the fitted curves to reflect the settlement trend within 180 days. It is clearly noticed that the cumulative settlement increases with time but the growth rate gradually turns to be slower. The cumulative settlements captured by observation stake #1∼#4 at around 180 days are maintained at 3.12 mm, 0.53 mm, 6.25 mm and 6.44 mm respectively.

**Fig 6:**
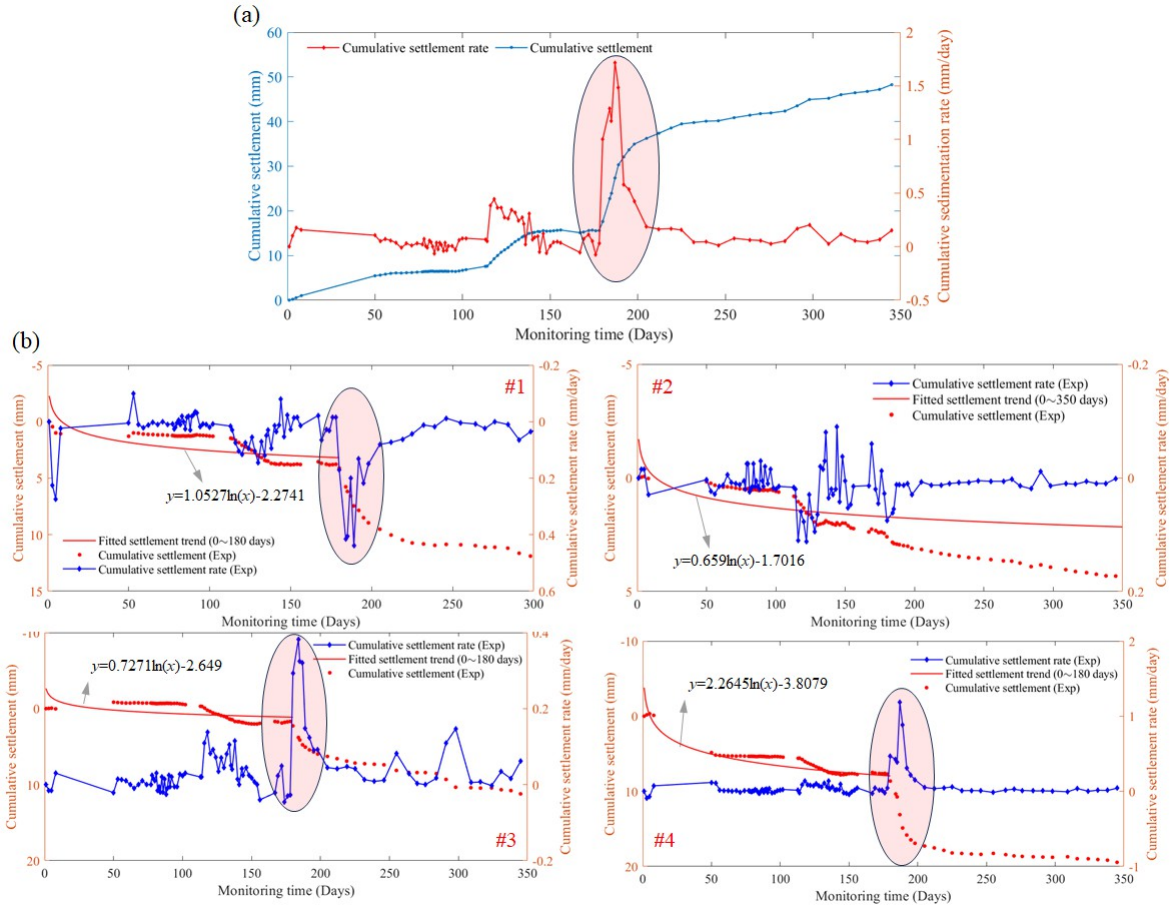
Cumulative settlement values and their rates obtained by the layered settlement gauge at observation stake 3 used for soft-soil roadbed monitoring. (a) The cumulative values of the four settlement gauges, (b) the experimental data obtained by settlement gauges #1, #2, #3 and #4, respectively.

Similarly, the experimental study has been conducted on observation point 3, which is located on one side of the highway, as shown in Fig 3. The experimental results are presented in Fig 6. To observe the pattern of settlement change for soft-soil roadbed highway over a longer period of time, specifically, the observation period is extended to 350 days. The settlement trend on observation point 3 within the previous 180 days is consistent with Fig 5. And there is an abnormal settlement phenomenon around 180 days, where the settlement rate suddenly increases and then levels off again. This anomalous settlement occurred in the soil layer where settlers #1, #3 and #4 located. The cumulative settlement values obtained for settlement gauges #1, #3 and #4 at 180 days are 4.11 mm, 2.27 mm and 8.64 mm respectively, whereas when the observation time reaches 200 days, the cumulative settlement values recorded are 8.95mm, 5.98 mm and 16.92 mm, which are all approximately doubled within 20 days. While the soil layer where settler #2 located did not experience a sudden shift in displacement.

**Fig 7:**
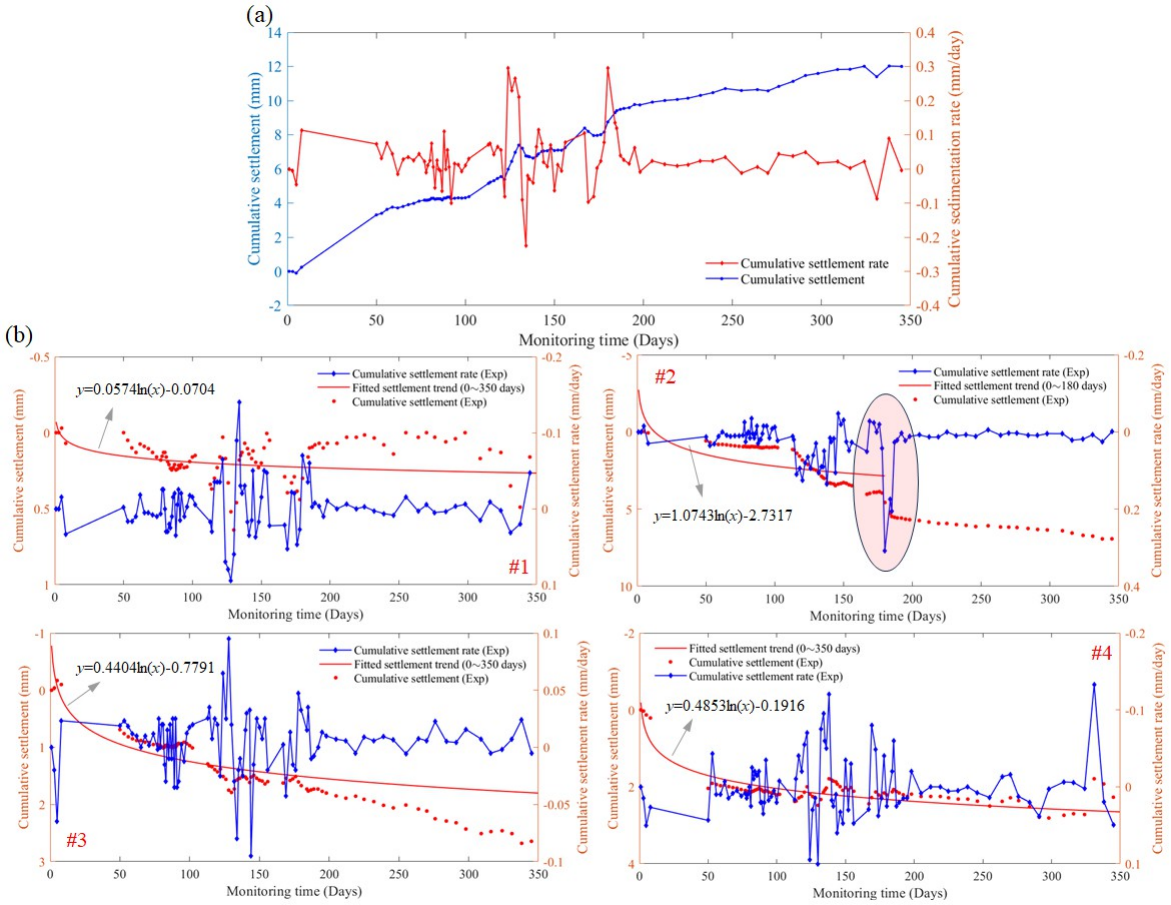
Cumulative settlement values and their rates obtained by the layered settlement gauge at observation stake 5 used for soft-soil roadbed monitoring. (a) The cumulative values of the four settlement gauges, (b) the experimental data obtained by settlement gauges #1, #2, #3 and #4, respectively.

As shown in Fig 7, observation point 5 on the other side of highway showed the opposite characteristics of Fig 6, where the abnormal settlement occurred in the soil layer where settler #2 located. The cumulative settlement measured by settlement gauge #2 at about 180 days is 4.57 mm, and after 20 days this value reaches 5.69 mm. While the settlement values in the soil layer where settlers #1, #3 and #4 installed increased with the increasing observation time, and the growth rate became slower. However, a special phenomenon of disturbance for the settlement rate is observed in the period of 110 to 180 days for these three settlers.

**Fig 8:**
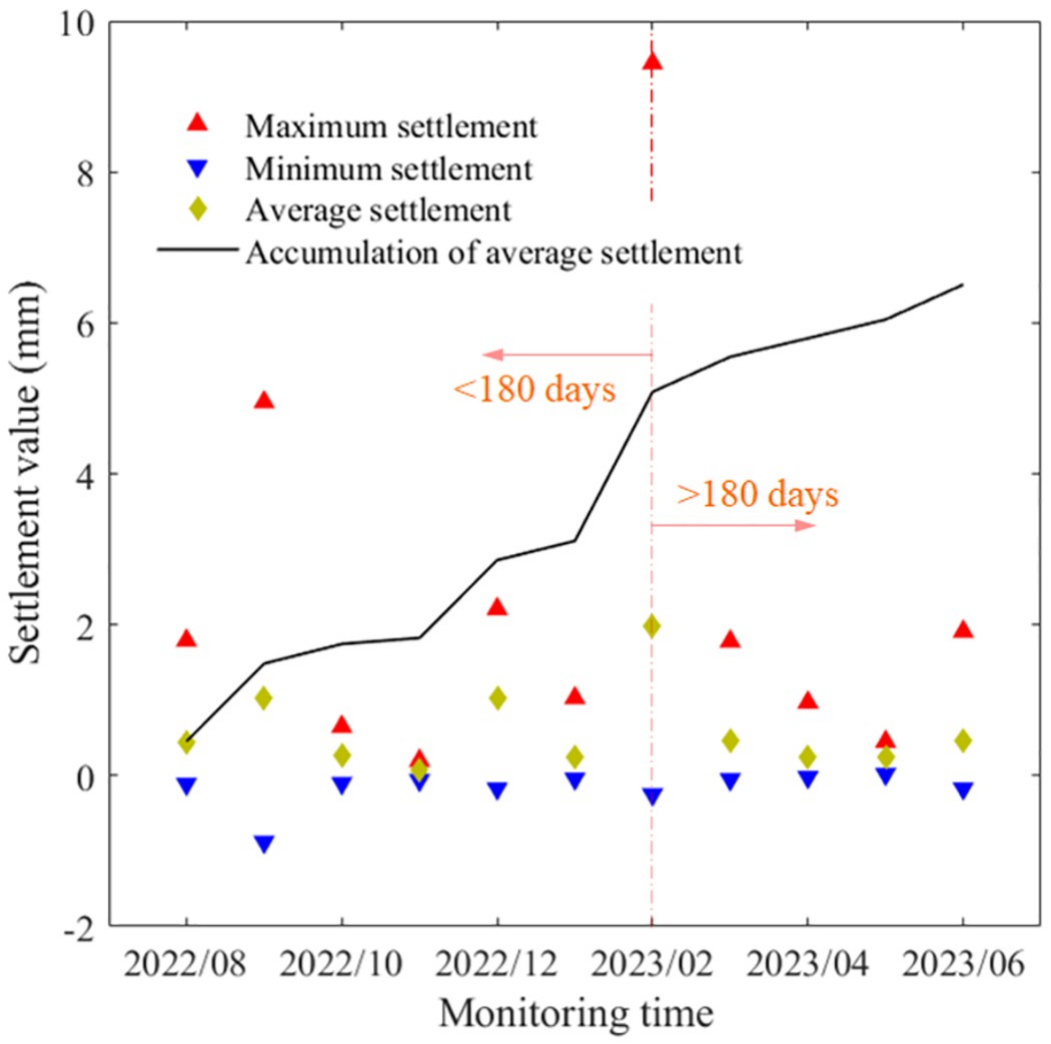
Maximum and minimum values, average and cumulative average of roadbed settlement obtained from all settlement gauges for section K114+340.

The maximum, minimum, average and cumulative average values of roadbed settlement obtained from all settlement gauges at section K114+340 are further tabulated as shown in Fig 8. It can be clearly observed that the maximum settlement value occurs at 180 days after the initial observation, with a maximum value of 9.44 mm. While the roadway settlement in this section increases with the increase of observation time, and the settlement rate tends to be slightly larger until 180 days, but then it gradually decreases again. This phenomenon is consistent with the combined view of Figs 5–7.

### Analysis for high slope monitoring

The focus of monitoring for the high and steep slope is the deformation measurements of platforms on slope. Taking K44+700 section of Jiuzhi-Markang highway construction project as an example, *X*, *Y* and *Z* displacement measurements were carried out by using total station at the positions of observation stakes 2, 3, 4 and 5 as shown in Fig 4, and aforementioned observation piles are located at the first, second, third and fourth level of slope platforms respectively.

**Fig 9:**
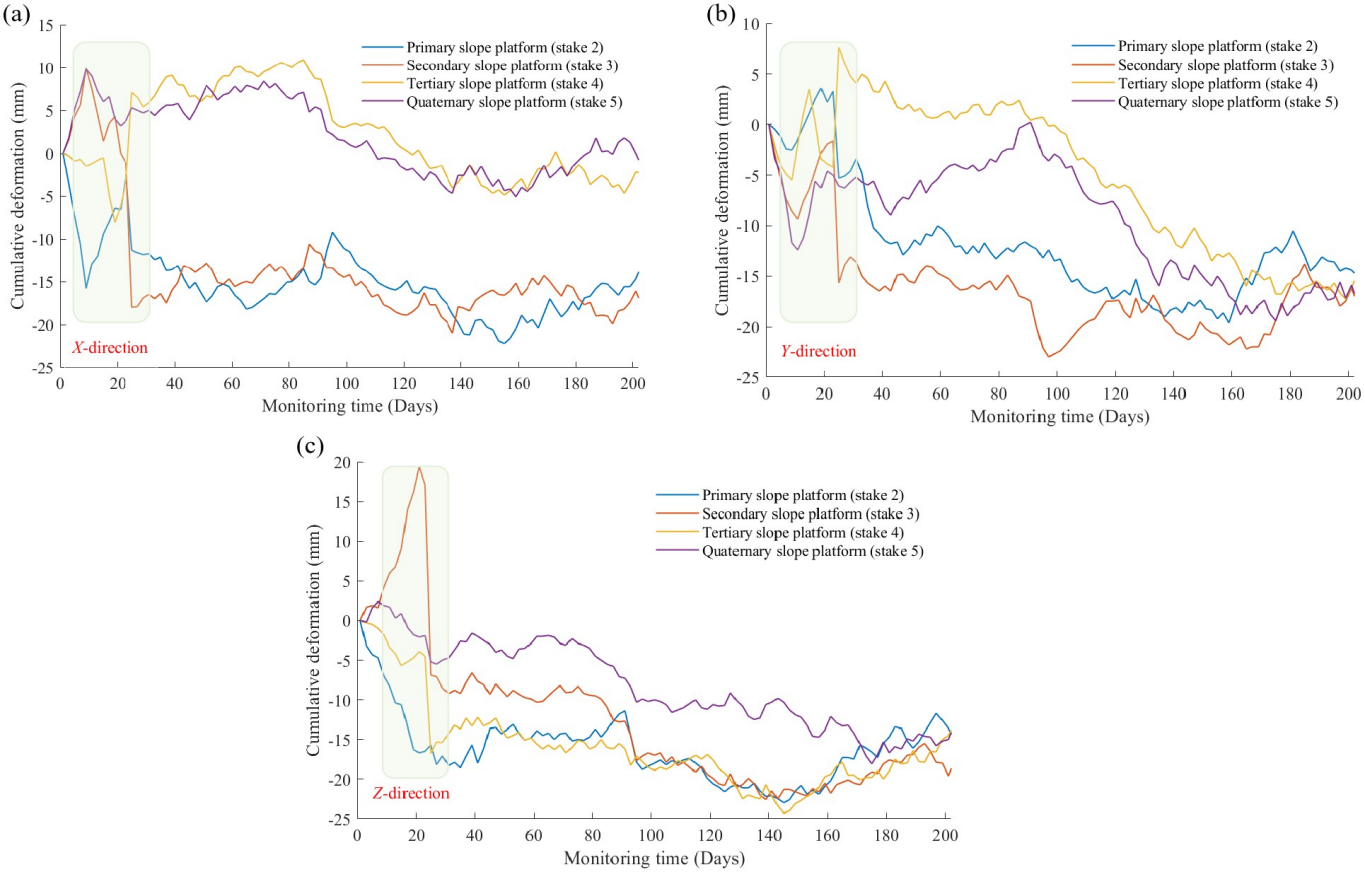
Curves of cumulative displacements with time in (a) *X*-direction, (b) *Y*-direction, and (c) *Z*-direction for primary, secondary, tertiary, and quaternary slope platforms captured by the total station at observation stakes 2, 3, 4, and 5.

Fig 9 illustrates the cumulative displacements of the first, second, third and fourth level of slope platforms in *X*, *Y* and *Z* directions captured by the total station. The cumulative displacements in each direction at all observation points have a general tendency to increase with increasing observation time. It is worth noting that this trend was broken at observation times of 10 to 30 days, and an abrupt reversal of deformation is captured by measuring equipment. This phenomenon was particularly evident in *Z* direction of the second slope platform. Furthermore, for in-plane displacements (*X* and *Y* directions), the cumulative deformations occurring at the higher elevation slope platforms (the first and second platforms) are always greater than the other two.

## Conclusions

The paper proposes in detail a way to monitor soft-soil roadbed settlement and high-slope deformation, to ensure the construction safety, efficiency and quality of highway construction, which is analyzed in conjunction with the experimental monitoring data. The layered settlement gauge and total station are employed to measure soft soil roadbed settlement (highway center axis and both sides) and high slope deformation (each platform of the slope), respectively. The main conclusions are shown as follows:

1. For highway with soft soil roadbed, the cumulative settlement and deformation on both sides will increase as the observation time increases. However, an abnormal settlement phenomenon was observed when monitoring time reached 180 days, and the rate of highway settlement accelerated unnaturally, while the location where this settlement acceleration occurred was diametrically opposite on both sides of the highway. For observation stake 3 on one side of the highway, the cumulative settlement values for settlement gauges #1, #3, and #4 are 4.11 mm, 2.27 mm, and 8.64 mm, respectively, over 180 days. Whereas the cumulative settlement values recorded when the observation period reached 200 days are 8.95 mm, 5.98 mm, and 16.92 mm, respectively, which are all approximately doubled over a 20-days period. For observation stake 5 on other side of the highway, the cumulative settlement value measured by gauge #2 was 4.57 mm around 180 days and it reached 5.69 mm after 20 days.
2. For highway with high slope, anomalous deformations of slope platform occur in *X*, *Y* and *Z* directions during 10 to 30 days of observation. Moreover, the in-plane cumulative deformations experienced by the slope platforms at higher elevations are always larger.

The findings can master the change pattern of settlement and lateral movement for highway in soft soil roadbed and high slope. And the way of monitoring the frozen-soil slopes located in high altitude areas can be specifically explored.

## Supporting information

S1 Data (XLS)

S1 File Settlement data obtained from real-time monitoring, descriptive conditions and displacement trend changes. (DOCX)
