# Supplementary material for: Monitoring and stability analysis of roadbed & high slope prior to highway construction
Source: PLoS One. 2024 Jun 17;19(6):e0303860. doi: 10.1371/journal.pone.0303860 (PMC11182516; doi:10.1371/journal.pone.0303860)
Supplement: S1 File — (DOCX) [file pone.0303860.s002.docx]

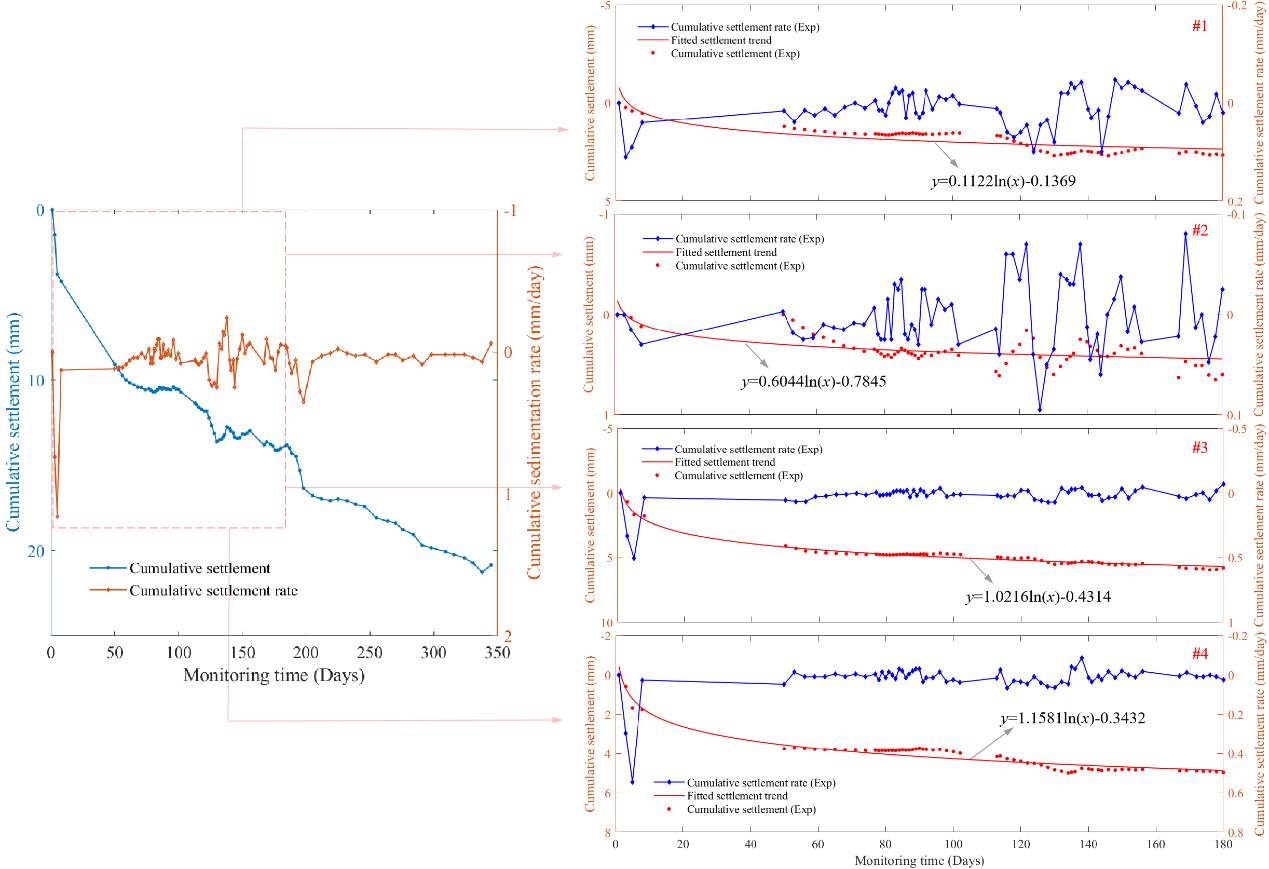


Cumulative settlement values and their rates obtained by the layered settlement gauge at observation stake 4 used for soft-soil roadbed monitoring. The left graph shows the cumulative values of the four settlement gauges, and the right graph shows the experimental data obtained by settlement gauges \#1, \#2, \#3 and \#4, from top to bottom, respectively.


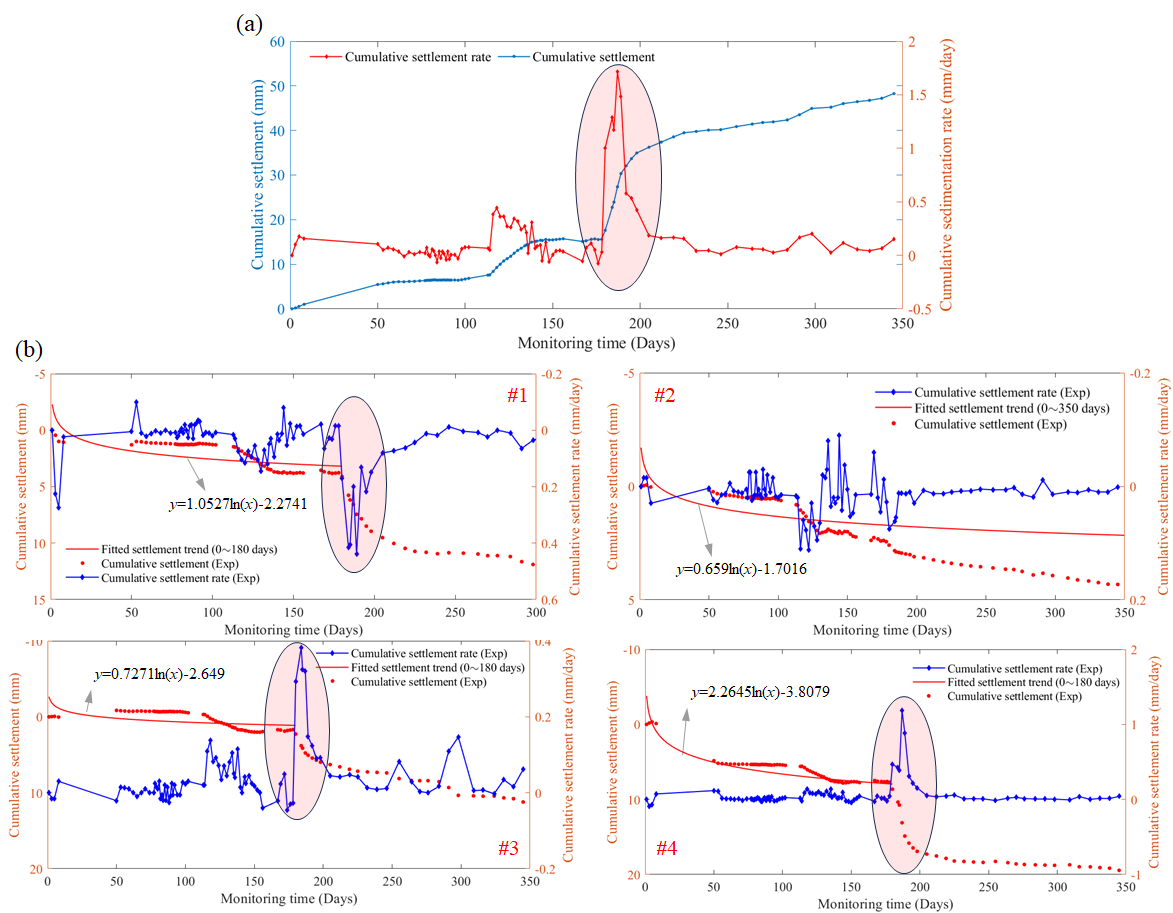


Cumulative settlement values and their rates obtained by the layered settlement gauge at observation stake 3 used for soft-soil roadbed monitoring. (a) The cumulative values of the four settlement gauges, (b) the experimental data obtained by settlement gauges \#1, \#2, \#3 and \#4, respectively.


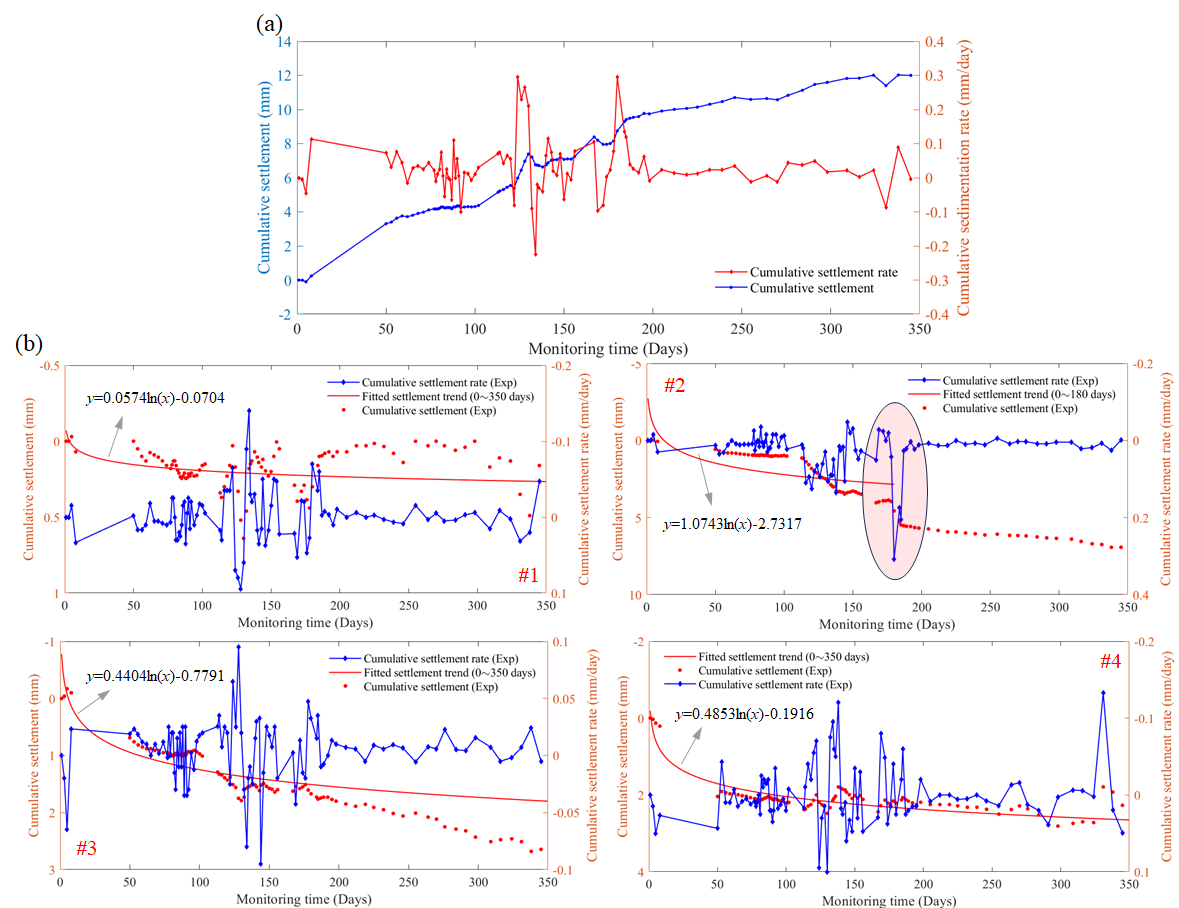


Cumulative settlement values and their rates obtained by the layered settlement gauge at observation stake 5 used for soft-soil roadbed monitoring. (a) The cumulative values of the four settlement gauges, (b) the experimental data obtained by settlement gauges \#1, \#2, \#3 and \#4, respectively.


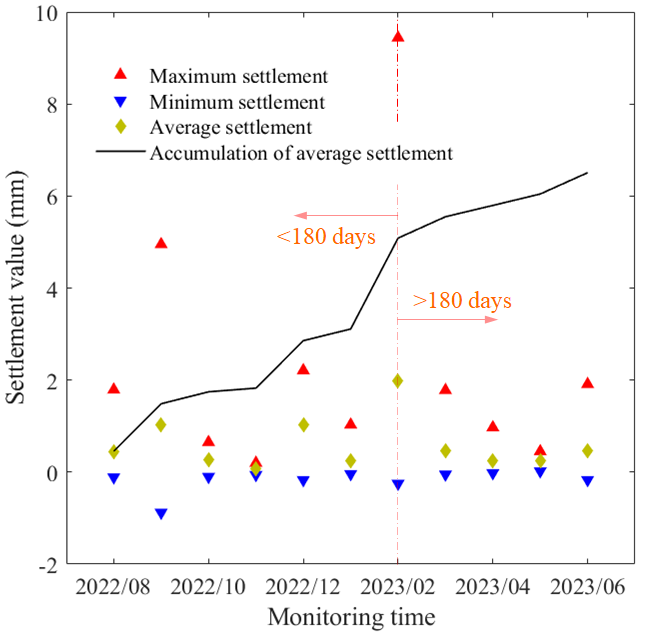


Maximum and minimum values, average and cumulative average of roadbed settlement obtained from all settlement gauges for section K114+340.


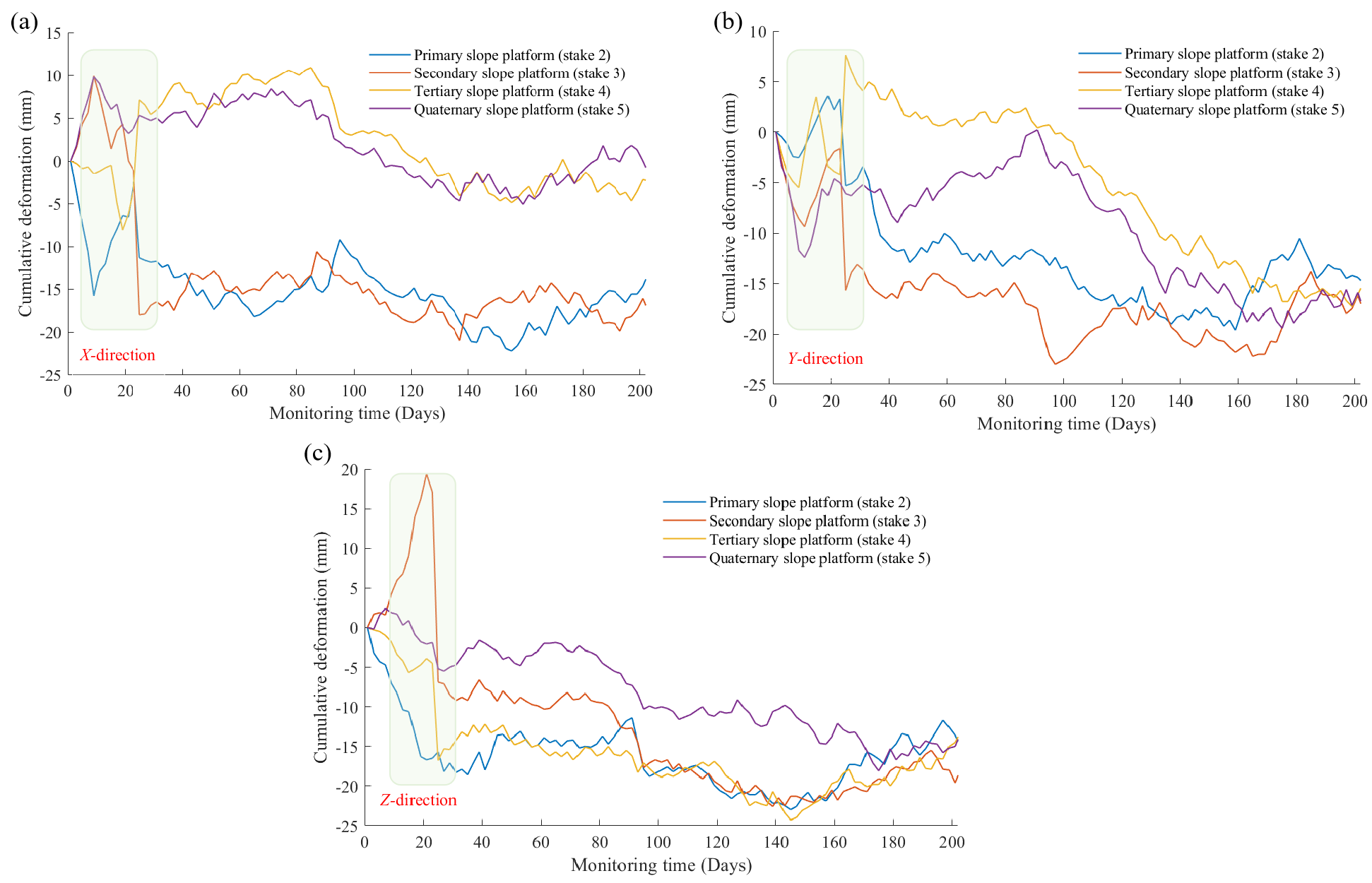


Curves of cumulative displacements with time in (a) X-direction, (b) Y-direction, and (c) Z-direction for primary, secondary, tertiary, and quaternary slope platforms captured by the total station at observation stakes 2, 3, 4, and 5.
